# Can mother-to-child transmission of HIV be eliminated without addressing the issue of stigma? Modeling the case for a setting in South Africa

**DOI:** 10.1371/journal.pone.0189079

**Published:** 2017-12-08

**Authors:** Holly J. Prudden, Matthew Hamilton, Anna M. Foss, Nicole Dzialowy Adams, Melissa Stockton, Vivian Black, Laura Nyblade

**Affiliations:** 1 Department of Global Health and Development, Faculty of Public Health and Policy, London School of Hygiene and Tropical Medicine, London, United Kingdom; 2 Avenir Health, Washington, DC, United States of America; 3 Communicable Disease Branch, Department of Health and Human Services, Raleigh, North Carolina, United States of America; 4 RTI, International and HP+, Washington, DC, United States of America; 5 Wits Reproductive Health and HIV Institute and Clinical Microbiology and Infectious Diseases, Faculty of Health Sciences, University of the Witwatersrand, Johannesburg, South Africa; University of Toronto Dalla Lana School of Public Health, CANADA

## Abstract

**Background:**

Stigma and discrimination ontinue to undermine the effectiveness of the HIV response. Despite a growing body of evidence of the negative relationship between stigma and HIV outcomes, there is a paucity of data available on the prevalence of stigma and its impact. We present a probabilistic cascade model to estimate the magnitude of impact stigma has on mother-to-child-transmission (MTCT).

**Methods:**

The model was parameterized using 2010 data from Johannesburg, South Africa, from which loss-to-care at each stage of the antenatal cascade were available. Three scenarios were compared to assess the individual contributions of stigma, non-stigma related barriers, and drug ineffectiveness on the overall number of infant infections. Uncertainty analysis was used to estimate plausible ranges. The model follows the guidelines in place in 2010 when the data were extracted (WHO Option A), and compares this with model results had Option B+ been implemented at the time.

**Results:**

The model estimated under Option A, 35% of infant infections being attributed to stigma. This compares to 51% of total infections had Option B+ been implemented in 2010. Under Option B+, the model estimated fewer infections than Option A, due to the availability of more effective drugs. Only 8% (Option A) and 9% (Option B+) of infant infections were attributed to drug ineffectiveness, with the trade-off in the proportion of infections being between stigma and non-stigma-related barriers.

**Conclusions:**

The model demonstrates that while the effect of stigma on retention of women at any given stage along the cascade can be relatively small, the cumulative effect can be large. Reducing stigma may be critical in reaching MTCT elimination targets, because as countries improve supply-side factors, the relative impact of stigma becomes greater. The cumulative nature of the PMTCT cascade results in stigma having a large effect, this feature may be harnessed for efficiency in investment by prioritizing interventions that can affect multiple stages of the cascade simultaneously.

## Introduction

Stigma and discrimination stigma continue to undermine the effectiveness of the HIV response and challenge the achievement of global goals. Such goals include the 90-90-90 targets [1], the elimination of infant (mother-to-child) HIV infections, keeping mothers alive [2] and achieving an AIDS-free generation[3, 4]. Despite the long-term and widespread acknowledgment of the negative role of stigma on the HIV response[5–7], a growing body of evidence of the negative relationship between stigma and specific outcomes including HIV testing, linkage to care, prevention of mother-to-child transmission (PMTCT) and adherence[8–12], there is still a paucity of national or sub-national data available on the drivers and prevalence of stigma. In addition, the magnitude of the effect of stigma on key outcomes, and hence of the potential benefits of stigma-reduction remains unclear[13].

Here, we present a probabilistic mathematical model designed to estimate the potential magnitude of the impact of stigma on just one outcome—mother-to-child transmission (MTCT) of HIV. This exploratory work is intended to demonstrate the potential of modeling as a method for examining the interactions between stigma and key HIV outcomes.

## Background

Since the UNAIDS Global Plan set goals for the elimination of MTCT and ‘keeping mothers alive’[2], significant progress has been made to identify and expand successful interventions for the prevention of mother-to-child transmission of HIV[2, 14]. The number of children newly infected fell from 270,000 in 2009 [230,000–330,000] to 110,000 [78,000–150,000] in 2015 worldwide–a 60% decrease[15, 16]. Despite this progress, in seven of the 21 Global Plan priority countries new vertical infections among children have declined by less than 30% since 2009[15]. Furthermore, in 2015 MTCT rose from 4.9% at six weeks to 8.9% at the end of breastfeeding—well above the 2% defined as the threshold for elimination–while at least a quarter of pregnant women were not started on lifelong antiretroviral therapy[16].

Accelerating progress towards elimination of MTCT and retaining women on lifelong treatment post-partum will require not only continued expansion of the supply of PMTCT and antiretroviral (ARV) treatment programs, but also an analysis of and response to demand-side barriers to access and adherence, including stigma[4–6]. In addition, as countries make progress towards addressing supply-side issues and strengthening health delivery systems, the relative role of structural factors, like stigma, will become more salient. As Watts et al. (2010) have demonstrated, the relative role of stigma on MTCT is greater in high than in low functioning PMTCT programs [17]. As countries approach elimination of MTCT, addressing stigma could be the key to making the final push to achieving elimination.

Stigma and discrimination within health services, families and the wider community have been documented as significant demand-side social barriers to PMTCT[9, 12, 18–20]. HIV-related stigma is a social process of devaluation of people either living with or associated with HIV that culminates in discrimination[21]. Discrimination (sometimes referred to as enacted stigma) is the unfair and unjust treatment of an individual based on his or her real or perceived HIV status[22]. Stigma is a barrier to PMTCT as it can keep women from seeking HIV testing, linking to and remaining in care or adhering to treatment. Stigma can manifest in many forms, from physical or social exclusion to verbal or physical abuse and can occur across all spheres of a woman’s life, from within the family, to at the health center. Both the experience or the anticipation (fear of) stigma act as barriers to testing, linkage to care and adherence.

**Fig 1:**
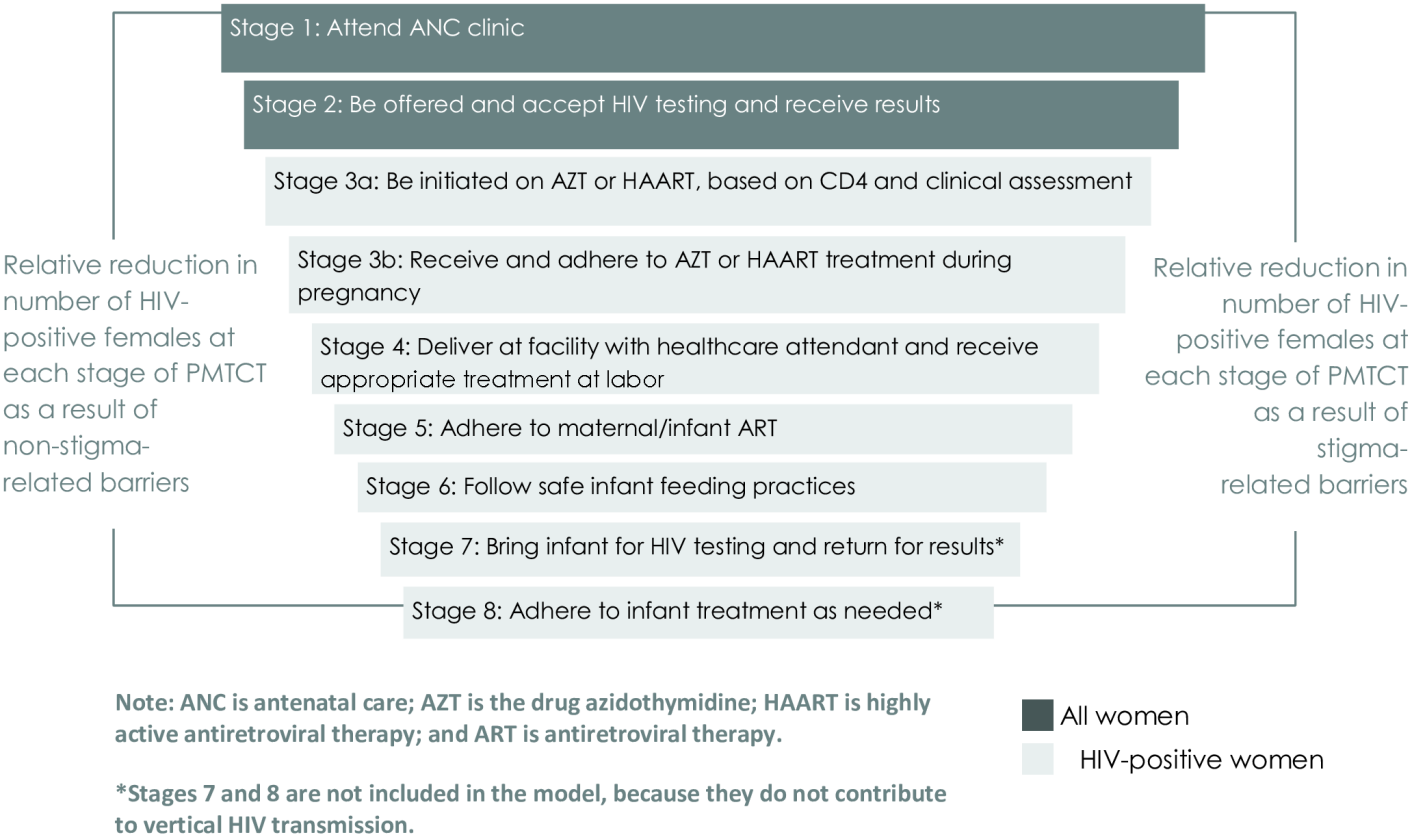
The PMTCT program cascade. Adherence at each stage is estimated to provide a cumulative reduction in the likelihood of the infant acquiring HIV, from a 30% chance of transmission in the total absence of PMTCT, to a 2% chance when all stages are adhered to.

The aim of this modelling exercise is to explore the potential impact of stigma on the uptake of and retention in HIV care during pregnancy and postpartum. This process is often conceptualized as a ‘cascade’, which describes the series of events, behaviors, and interventions that begin with women’s first appearance at antenatal care (ANC) and continue through post-delivery services and infant feeding. Fig 1, illustrates the cascade in a simplified form, highlighting the cumulative loss of women who leave the cascade at each step, either from stigma barriers[12] or barriers not related to stigma. If all recommended steps of the process are completed, the average risk of MTCT is reduced from approximately 30% to less than 2%[23, 24].

## Methods

We present a mathematical model designed to estimate the impact of stigma on PMTCT of HIV and to demonstrate the potential impact of reductions in stigma on the number of new infant HIV infections. Mathematical modeling offers a way to circumvent the challenges involved in empirically measuring, for example, the effect of stigma on transmission. This is achieved by using data from the setting and the literature, to estimate the proportion of infant infections that would occur if action were taken to reduce stigma, compared with model estimates if no such action were taken. These estimates are used to calculate potential reductions in the mother to-child transmission rate that would occur if preventative action to reduce stigma were to be implemented. Building on the modeling work of Watts *et al*. (2010)[17], we adapted their model based on the drug nevirapine (NVP) to reflect the World Health Organization (WHO) (2010) guidelines for PMTCT treatment and applied this to a setting in South Africa, modelling the PMTCT process as a probabilistic cascade with loss-to-care at each stage. Given that data for parameterizing this model were extracted from the District Health Information System (DHIS) at the time that the 2010 guidelines were in place, the model follows these guidelines (WHO Option A), as opposed to Option B+, now employed by South Africa and 21 of the 22 priority countries[25]. Therefore, we also include a scenario using the current WHO B+ guidelines in order to compare outcomes.

### Setting

We selected Johannesburg, South Africa, as the setting for this work for several reasons. South Africa had been implementing the WHO revised PMTCT guidelines since April 2010, long enough to provide appropriate data to support parameterization of the model at the time of data extraction. Secondly, South Africa has a national data collection process that documents participation in PMTCT at the district level through the first four stages of the PMTCT cascade[26], which allowed us to directly estimate the number and percentage of women lost to care at each stage. Various reports across different sites in South Africa have documented high levels of stigma, including one qualitative study from Johannesburg [27]. Unfortunately, though, no quantitative data specific to Johannesburg were available.[28–31].

The 2010 South Africa PMTCT guidelines largely resembled “Option A” [32] and are highlighted in Table 1 [33]. These guidelines were revised in 2013 to offer Option B, and again in 2015 as part of the push toward “Option B+”[32], under which all women, regardless of CD4 cell count would initiate ARVs upon diagnosis and continue treatment throughout the duration of breastfeeding and then for life [34]. Option B+ offers triple ART drugs that continue for life, with the infant receiving NVP or AZT from birth through age 4–6 weeks regardless of feeding method.

**Table 1:** WHO 2010 guidelines for South Africa PMTCT.

Stages in Cascade	2010 South Africa PMTCT Guidelines
**Stage 1:** **Women accessing ANC services**	Provide PMTCT services at antenatal clinics (integrate ART and ANC services)
**Stage 2:** **Women offered and accept HIV test and counseling**	ANC clinic provides routine HIV test and counseling to all women attending clinic
**Stage 3:** **Women already on ART before 1**^**st**^ **ANC visit**	If regimen includes EFV, switch to NVP if in first trimester only; continue regimen throughout labor/delivery; continue lifelong ART after pregnancy
**Stage 3:** Women who test positive and receive results*	After CD4 test: >350 cells/mm^3^-initiate AZT at 14 weeks gestation <350 cells/mm^3^-initiate HAART as soon as results are received (TDF + 3TC/FTC + NVP)
**Stage 4:** **Labor and delivery**	Women on AZT: receive sdNVP and AZT 3hrly until delivery; stat TDF and FTC after delivery Women on HAART: continue with HAART regimen; do not receive additional drugs during this time
**Stage 5:** **Infant treatment after birth**	Women on AZT: receive sdNVP within first 72 hours; then 6 week dose of NVP (or for duration of breastfeeding if longer than 6 weeks) Women on HAART: receive sdNVP within first 72 hours receive 6 week dose of NVP
**Stage 6:** **Infant feeding recommendations**	Counsel mothers on safe feeding practices-exclusive breastfeeding or exclusive formula feeding for 6 months (South Africa starting to focus solely on exclusive breastfeeding)

### Model structure

The model follows a fictional cohort of 100,000 pregnant women through the PMTCT cascade. Each stage of the cascade specifies an action resulting in either retention or loss from the cascade—a binary outcome that depends on exposure to stigma, as well as other, non-stigma-related barriers. The model includes the first six of eight steps in the 2010 South Africa PMTCT guidelines (Option A)[33]. The final two stages of the cascade—infant testing and treatment of HIV positive infants—are not included because they do not directly affect MTCT of HIV (Fig 1).

We consider three scenarios in the model: *status quo* including stigma and non-stigma-related barriers; *low stigma including* only non-stigma-related barriers; and *ideal* with no barriers to access. The status quo scenario is based on data taken from the PMTCT cascade that is characteristic of Johannesburg. We take Johannesburg as representative of a setting in which both stigma and non-stigma related barriers are likely to exist within the treatment cascade. Examples of stigma-related barriers tend to be demand-side barriers, such as fear of testing for HIV, and fear of disclosing status and initiating treatment. Non-stigma-related barriers more generally are supply-side barriers, such as accessing transport services, drug stock-outs, or the cost of attending a facility because of its location. The low stigma scenario, therefore, reflects an environment in which stigma have little or no impact on women’s behavior and decision-making, and those barriers that remain are predominantly supply-side barriers. In order to estimate this scenario we conducted a literature review to identify studies that estimated the percentage of women retained in care at each stage of the cascade. We made the assumption that under study conditions women have greater support in accessing care (compared with the status-quo which is outside of studies) and therefore levels of stigma are lower. In the ideal scenario stimga do not exist, all non-stigma-related barriers are removed, and all HIV-positive women receive and adhere to the most effective available treatment, highly active antiretroviral therapy (HAART). The few transmission events in the ideal scenario occur because even HAART is not 100% effective in preventing vertical transmission (“drug failure”).

**Fig 2:**
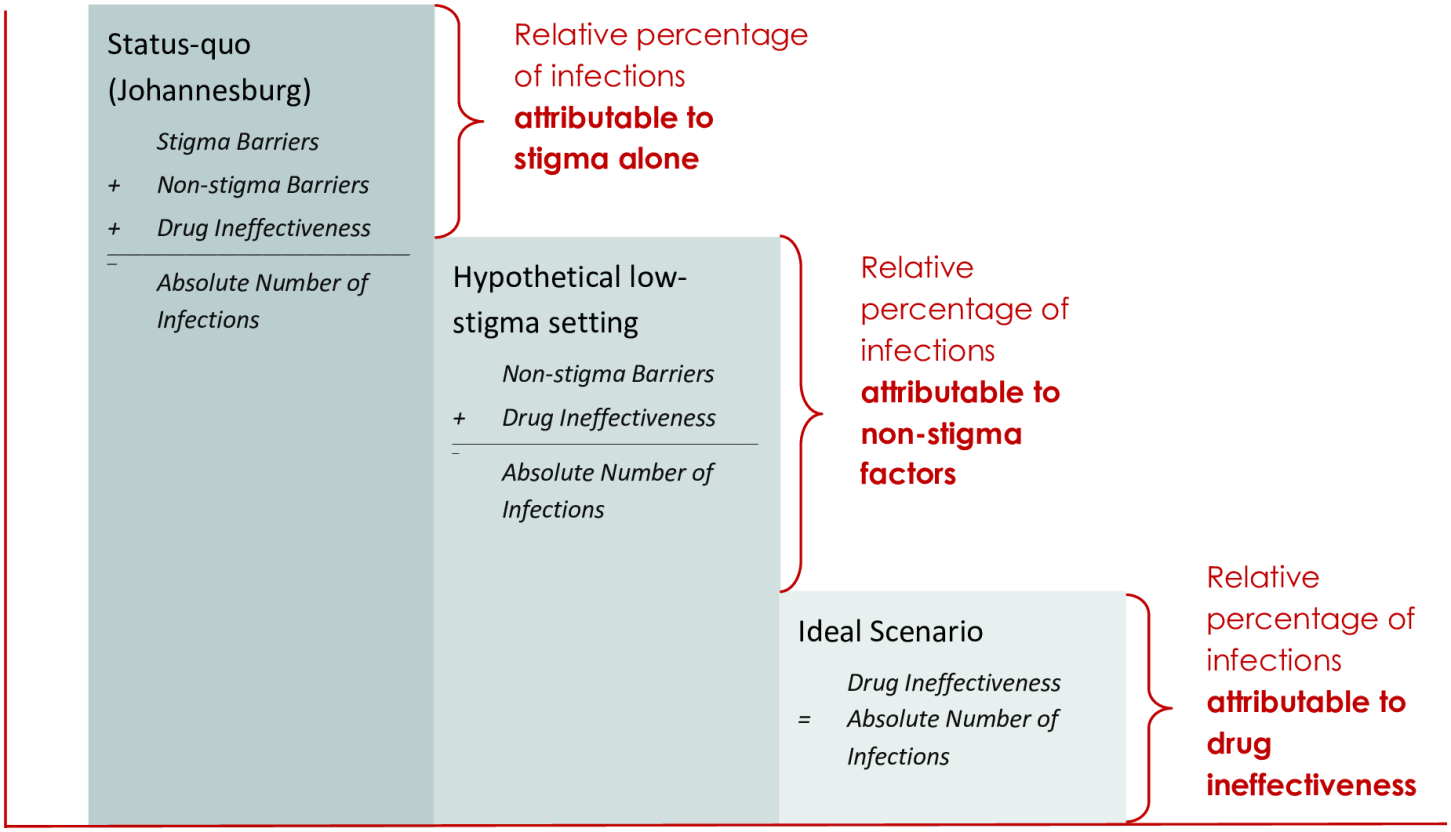
Conceptual diagram. Conceptual diagram of the model representing relative percentages of infant infections that are attributable to stigma-related and non-stigma-related barriers and to drug ineffectiveness.

The structure of the cascade is the same for all three scenarios; scenarios are distinguished only by the event probabilities at each stage of the cascade. By comparing transmission across these three scenarios, we can estimate the average mother-to-child transmission rate in the status quo scenario that are attributable to stigma-related barriers, non-stigma-related barriers, and to drug failure in the absence of any barriers (Fig 2). The number of infections attributable to stigma-related barriers is the difference between infections in the status-quo and low stigma scenarios. The number attributable to other, non-stigma-related barriers is the difference between infections in the low stigma and ideal scenarios. Finally, the number of infections remaining in the ideal scenario are those attributable to drug failure.

**Fig 3:**
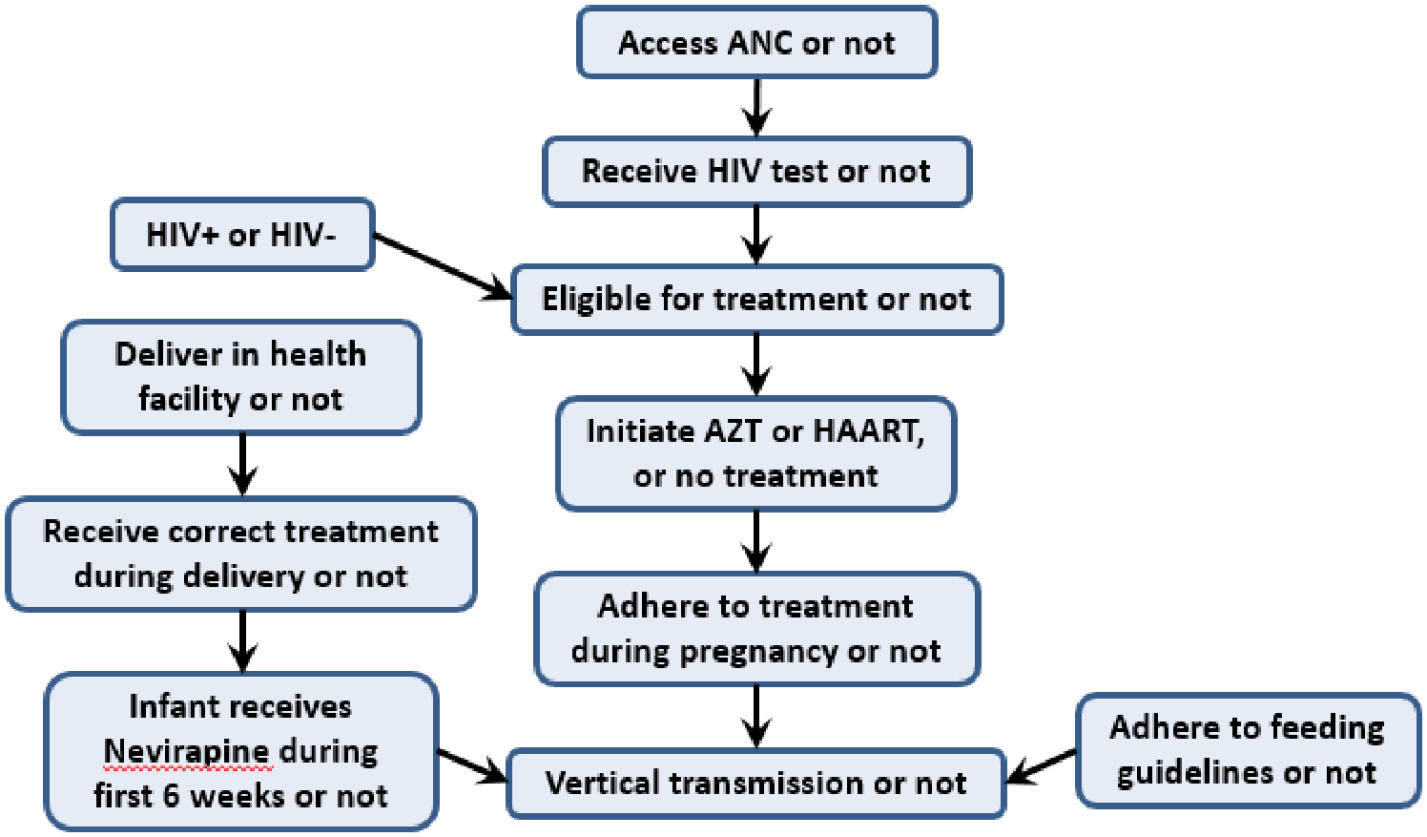
Conditional dependency relationships among model variables.

The key outcome of interest in the model is the average mother-to-child transmission rate due to stigma. The model represents the PMTCT cascade in terms of 11 variables: 10 variables that influence transmission, and transmission itself. Fig 3 graphically depicts the dependency relationships among these variables that define the model’s structure. The treatment initiation variable can take on one of three possible values, reflecting initiation on zidovudine (AZT), HAART, or no treatment. Each of the other variables have just two possible values. The value of each variable for each woman in the cohort is determined by a conditional probability distribution, where the probability of each possible value is conditional on the values of the variables on which it depends. The specific probabilities used are determined from the available data from Johannesburg (DHIS data) and, where setting-specific data is lacking, through drawing on the broader literature (see Supplementary Materials). By modifying the probabilities associated with each variable, we can model both the influence of non-stigma related barriers and the effects of stigma-related barriers at each node.

#### Option A

The cascade begins when a woman accesses ANC services. Women who do not access ANC are lost from the cascade. Women who attend ANC may receive an HIV test. If a woman receives a test, and if she is HIV+, she is eligible for treatment and may initiate AZT (if CD4>200cells/ mm^3^) or HAART (if CD4 count<200cells/mm^3^), based on the 2010 South Africa PMTCT guidelines (see Table 1) which at the time followed the Option A programme, or she may fail to initiate treatment. If she initiates, she may or may not adhere to treatment throughout her pregnancy. Whether she adheres to treatment directly affects her probability of transmission, but all variables “upstream” in the cascade indirectly affect transmission by affecting whether she adhered to treatment or not. The probability of transmission also depends directly on whether the infant receives NVP for six weeks, and whether the mother adheres to feeding guidelines.

#### Option B+

In addition to modelling the treatment guidelines for Option A, which were in place in 2010 at the time of the data collection, we explored the case in which Option B+ was followed. This was in accordance with the new 2015 guidelines that recommend lifelong antiretroviral treatment for all pregnant and breastfeeding mothers living with HIV regardless of their CD4 cell count or WHO clinical stage. Treatment is maintained in the long-term after completion of delivery and breastfeeding. Thus we effectively modelled B+ as if it had been introduced into an environment characterized by the situation in 2010 Johannesburg. For the model structure, this meant that the AZT option was effectively removed, all women were placed on HAART after testing positive for HIV, and this treatment was maintained throughout their pregnancy, delivery, and breastfeeding of the infant. In this case our aim was to compare the relative impact of stigma, between Options A and Options B+.

### Model parameterization

The model parameters can be divided into two groups: 1) scenario probabilities that express the loss-to-care through the cascade as a result of stigma- and non-stigma related barriers, and 2) transmission rates that give the probability of transmission as a function of treatment status, use or non-use of infant NVP, and adherence to infant feeding guidelines. We obtained values for scenario probabilities for the number of females retained in care at each stage of the cascade from two main sources: (i) for the low-stigma scenario, a comprehensive literature review (111 articles) on both stigma and non-stigma related barriers to uptake and retention in PMTCT programs with a specific focus on South Africa and the East and Southern Africa region (see Supplementary Materials for search criteria and additional summaries of key article details), and (ii) for the status-quo scenario from the Johannesburg DHIS[26].

Table 2 shows the scenario probabilities for the percentage of women who are accessing PMTCT at each stage in the model, for status quo, low stigma and the ideal scenarios, for women who had AZT or HAART initiated at ANC. For example, according to the DHIS, 97.8% of women attending ANC were offered an HIV test, were tested and received their results; thus, the status quo scenario probability that a woman will test, conditional on her attendance at ANC, is 97.8%.

**Table 2:** Scenario probabilities for the percentage of women who are accessing PMTCT at each stage in the model, for ideal, low and status quo scenarios, for women who had AZT or HAART initiated with ANC.

		AZT Cascade		HAART Cascade		Sources*
Stage		Low stigma	Status quo	Low stigma	Status quo	
**1**	Percentage of women accessing ANC	99	97.8	99	97.8	[19, 20, 26, 35–38]
**2**	Percentage of women offered and accepting HIV test results	99.1	93.2	99.1	93.2	[3, 37, 39–46]
**3**	Percentage of eligible women initiated on treatment	95	92.3	80	48	[19, 26, 37, 47–51]
**3**	Percentage of women who adhere to treatment regimen	90	65	90	65	[3, 19, 51–54]
**4**	Percentage of women who give birth in a health facility or with trained assistants**	96	91.2			[19, 26, 36, 38, 42, 55]
**4**	Percentage of women who receive proper treatment at delivery**	90	86			[3, 56, 57]
**5**	Percentage of infants who take 6-weeks of NVP	94.1	81	94.1	81	[19, 26]
**6**	Percentage of women who exclusively breastfeed for 6 months	87	50	87	50	[3, 19, 37, 58–62]

*More detail on the literature sources and key points for both the stigma and non-stigma factors is found in the supplementary materials.

**Mothers who are receiving AZT require single-dose Nevirapine (sdNVP) prior to delivery, and then require Tenofovir (TDF) and Truvada (FTC), after delivery (Step 4, Table 1), which are required to be administered at a birthing facility. This is not the case for women on HAART.

For the second group of parameters the model incorporates 12 rates of vertical transmission, corresponding to each combination of three treatment regimens during pregnancy (AZT, HAART or none), infant adherence or non-adherence to NVP for six weeks post-delivery, and adherence or non-adherence to feeding guidelines (Table 3). These are drawn from a review of the current literature on transmission rates in various prevention scenarios, and include data both from within and outside South Africa. All of the studies from the literature that estimate transmission rates had either a three-month or six-month follow-up period to evaluate the effectiveness of PMTCT for different treatment scenarios.

**Table 3:** Transmission rates corresponding to each combination of 3 treatment regimens during pregnancy (AZT, HAART or none), infant adherence or non-adherence to NVP for 6 weeks post-delivery, and adherence or non-adherence to feeding guidelines.

Probability Transmission Estimate	Percentage	95% Confidence Interval	References*
Probability of vertical transmission in the absence of PMTCT and lack of adherence to infant feeding guidelines	30	25–35	[24, 63]
Probability of vertical transmission when mother is on AZT regimen, infant does not receive NVP, and there is no adherence to infant feeding guidelines	21	13–30	[64]
Probability of vertical transmission when mother receives no treatment, infant does receive NVP, and there is no adherence to infant feeding guidelines	13.6	12.9–15.8	Imputed**
Probability of vertical transmission when mother receives no treatment, infant does not receive NVP, and there is adherence to infant feeding guidelines	25.7	23.8–27.4	Imputed**
Probability of vertical transmission when mother receives no treatment, infant does receive NVP, and there is adherence to infant feeding guidelines	8.3	6–12.3	Imputed**
Probability of vertical transmission when mother is on AZT regimen, infant does not receive NVP, and there is adherence to infant feeding guidelines	18	12.4–23.5	[65]
Probability of vertical transmission when mother is on AZT regimen, infant receives NVP, and there is no adherence to infant feeding guidelines	9.5	6.7–13.5	[66]
Probability of vertical transmission when mother is on AZT regimen, infant receives NVP, and there is adherence to infant feeding guidelines	5.8	3.1–10.5	[66]
Probability of vertical transmission when mother is on HAART regimen, infant does not receive NVP, and there is no adherence to infant feeding guidelines	5.1	2.8–9.0	[67]
Probability of vertical transmission when mother is on HAART regimen, infant does not receive NVP, and there is adherence to infant feeding guidelines	2.9	1.9–4.4	[68]
Probability of vertical transmission when mother is on HAART regimen, infant receives NVP, and there is no adherence to infant feeding guidelines	4.8	2.9–8.0	[66]
Probability of vertical transmission when mother is on HAART regimen, infant receives NVP, and there is adherence to infant feeding guidelines	1.1	0.5–2.2	[69]

*Data extracted from southern African countries including South Africa.

**See Supplementary Materials.

Three of the 12 transmission rates were unavailable from the literature, corresponding to those women who did not initiate or adhere to any treatment during pregnancy, but who did either 1) give NVP, or 2) follow feeding guidelines, or 3) both. We estimated these three “missing” transmission rates among women not on treatment by reducing the base rate of transmission that is the transmission rate with no treatment, no NVP, and no adherence to feeding guidelines. More specifically, we reduced the base rate by the same relative proportion that obtained among women who adhered to AZT, and either 1) gave NVP, 2) followed feeding guidelines, or 3) both.

The model also uses the HIV prevalence [70] and the rate of infant mortality not attributable to HIV [71, 72] to ensure only deaths attributed to HIV are included in the estimates. This final parameter value was estimated using the under one year infant mortality rate, adjusted by subtracting the percentage of all those under one year whose death is attributed to HIV[71].

The three model scenarios share the same set of transmission rates, and are only distinguished by their different scenario probabilities, which characterize the levels of stigma and govern flow through the cascade (Fig 3). This differential flow through the cascade results in different weighted average transmission probabilities over the cohort for each scenario, leading to differences in the average mother-to-child transmission rate for each scenario.

### Uncertainty analysis

Aside from the detailed setting-specific data used to parameterize the model where possible, as is common in modelling analyses, many of the model inputs are our best estimates, based on the available literature. In order to address the associated uncertainty in these model inputs, we generated an ensemble of 100,000 fictional cohorts of 100,000 women each, with each cohort characterized by a different set of scenario probabilities and transmission rates sampled from interval estimates. The model was coded in MATLAB R2012b and the uncertainty analysis was performed by the same programme. Sampling of the parameter sets for the uncertainty analysis was performed using a Latin hypercube sampling technique [73]. For the uncertainty analysis, we estimated the variation in the average mother-to-child transmission rate for the three scenarios.

The interval estimates from which the sampling was done for the transmission rates are taken directly from the literature, usually 95% confidence intervals (CIs). We used a uniform distribution for transmission rates in order to be consistent with the standard interpretation of a confidence interval.

Scenario probabilities in the status quo scenario are empirical point estimates drawn from literature and Johannesburg-specific DHIS data, but do not come with empirical estimates of uncertainty such as CIs. Scenario probabilities in the low stigma scenario are conservatively imputed as point estimates, based on our exhaustive literature review. Since there were no empirically measured estimates for the scenario probabilities for either the status-quo or low-stigma scenario, we sampled from a normal distribution characterized by the mean as equal to the point estimate and a relatively large standard deviation of 0.1 (as all model parameters are scaled to the unit interval). We used a normal distribution for scenario probabilities so that the ensemble would contain relatively few cohorts characterized by unreasonably outlying combinations of scenario probability values. The resulting ensemble of fictional cohorts is weighted toward the DHIS data and estimates taken from the literature that best represent the low-stigma scenario but still includes significant variation in scenario probability values to represent the uncertainty in these estimates.

We repeated this process for the estimates generated by considering Option B+, to compare the uncertainty range with that for estimates under Option A.

## Results

**Fig 4:**
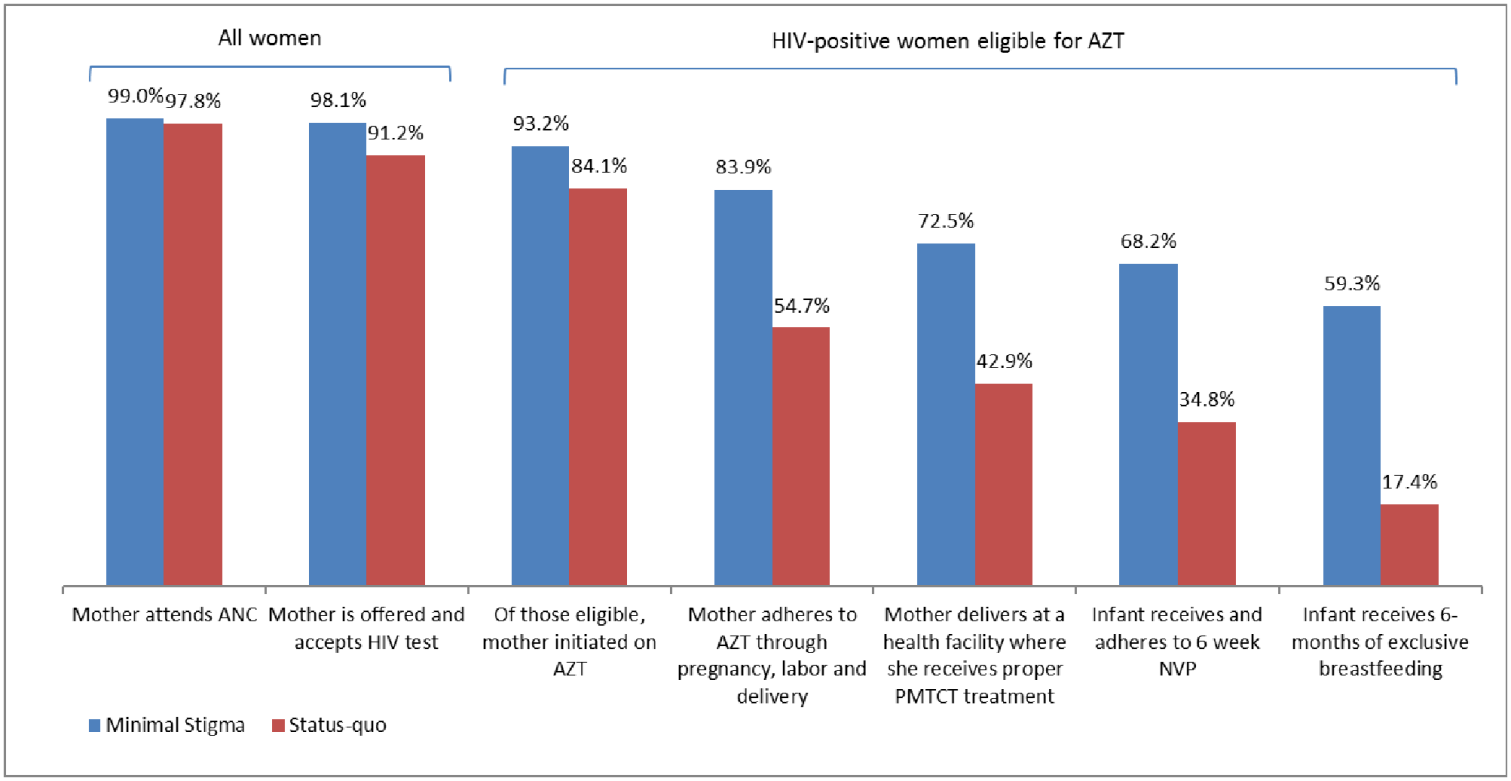
Percentage of HIV-positive women eligible for AZT prophylaxis who are cumulatively lost at each stage of the PMTCT cascade.

**Fig 5:**
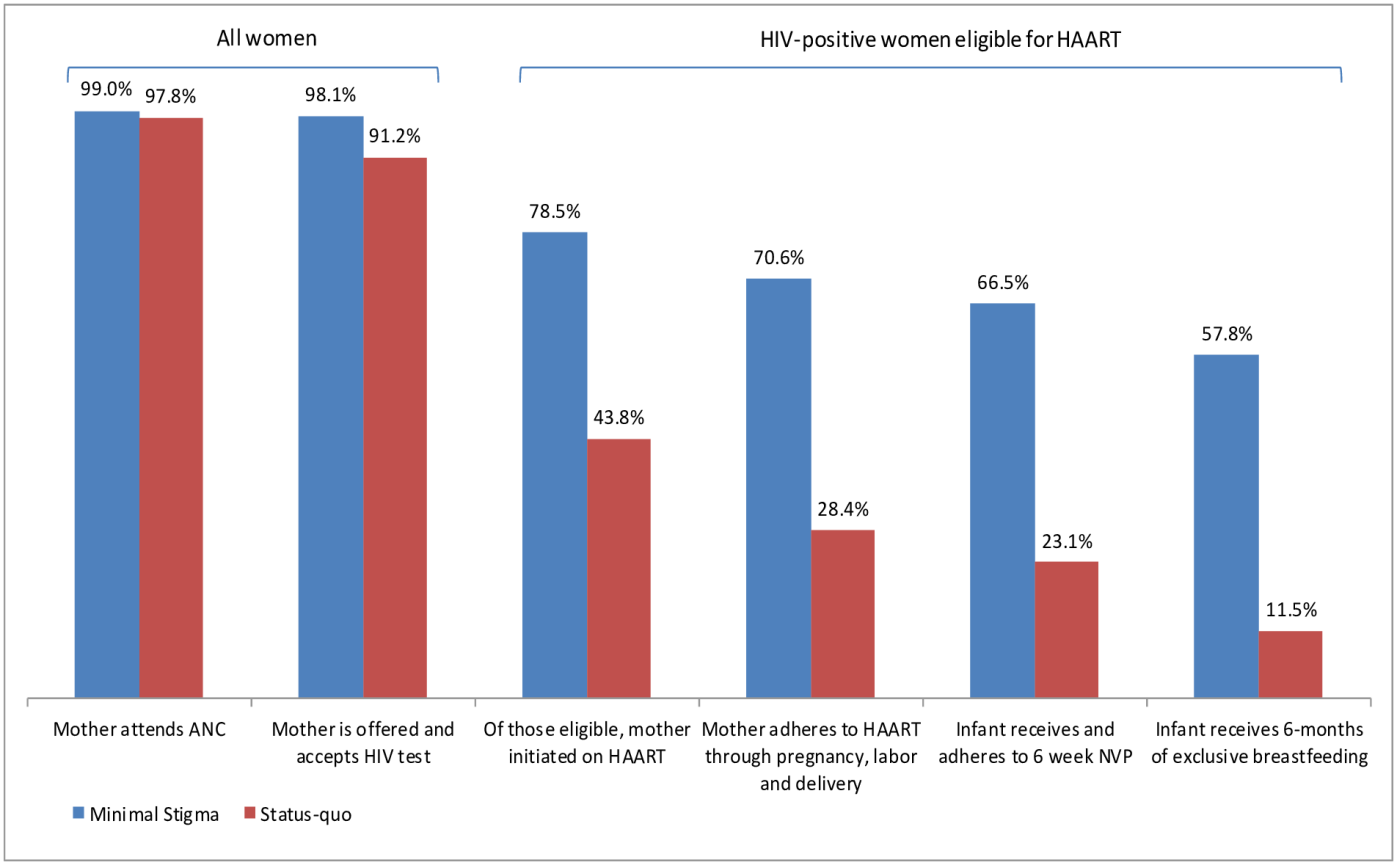
Percentage of HIV-positive women eligible for HAART who are cumulatively lost at each stage of the PMTCT cascade.

Figs 4 and 5 depict how flow through the cascade varies between the low stigma and status quo scenarios, as a result of variation in the percentage of women who are lost to the treatment cascade at each stage. The effect of stigma is to dramatically reduce the percentage of women receiving services and practicing the behaviors necessary to prevent infection at every stage of the PMTCT cascade.

**Fig 6:**
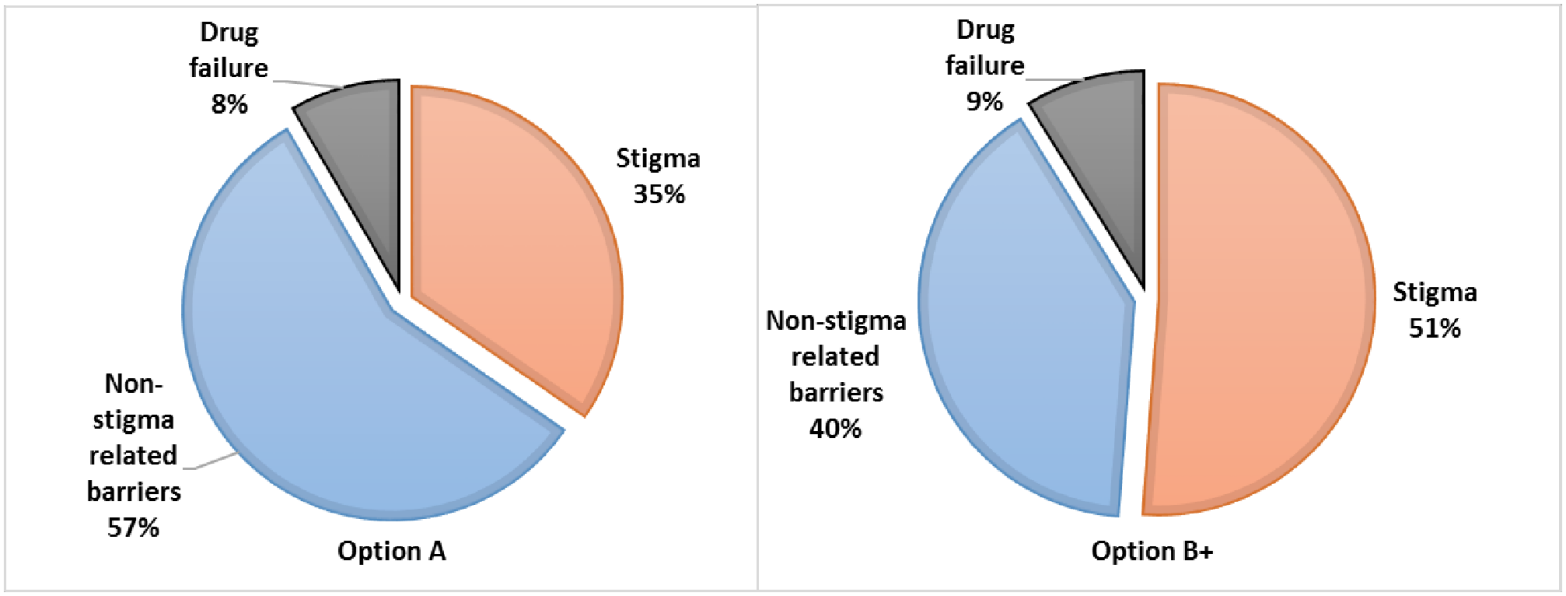
Distribution of infant infections (median values) as attributable to individual factors.

Fig 6 compares the distribution of infant infections attributable to drug-failure, non-stigma- related barriers and stigma-related barriers; for the Option A programme, which was implemented in 2010, and hypothetically for the case of Option B+ having been implemented that same year. In 2010, an estimated 35% of infant infections were attributed to stigma under Option A, compared to 51% if Option B+ had been implemented at that time. In Only 8% (Option A) and 9% (Option B+) of infant infections are attributed to drug ineffectiveness, with the trade-off in the proportion of infections being solely between stigma-related barriers and non-stigma-related barriers.

The total number of new infant infections is less with Option B+ than Option A since all women are offered HAART in Option B+, whereas in Option A women are offered either NVP or HAART, depending on CD4 cell count. For Option A, for the status quo scenario, the model projects 4,309 infant infections per 100,000 women, compared to 2,815 per 100,000 women for the low-stigma scenario. This represents an additional 1,495 infant infections that are estimated to occur primarily as a result of stigma, and translates to 35% of all infant infections being averted if stigma were eradicated in this setting. For Option B+, for the status quo scenario, the model projects 4,082 infant infections per 100,000 women, compared to 1,992 per 100,000 women for the low-stigma scenario, translating to 51% of all infant infections being averted if stigma were eliminated. Based on the data from South Africa (2010), a lower percentage of women were retained in the cascade for HAART (11.5%), compared to 17.4% for AZT. Since women on Option B+ take only HAART, whereas those on Option A predominantly take AZT (unless their CD4 count is <200cells/mm^3^), fewer women on Option B+ are retained in the cascade. Therefore, although HAART is a more effective treatment than AZT, the lower percentage of women who completed HAART means that the relative reduction in infant infections with Option B+ compared to Option A is not as pronounced as it would have been if retention in the cascade on HAART was the same as for those on AZT.

### Uncertainty analysis

**Fig 7:**
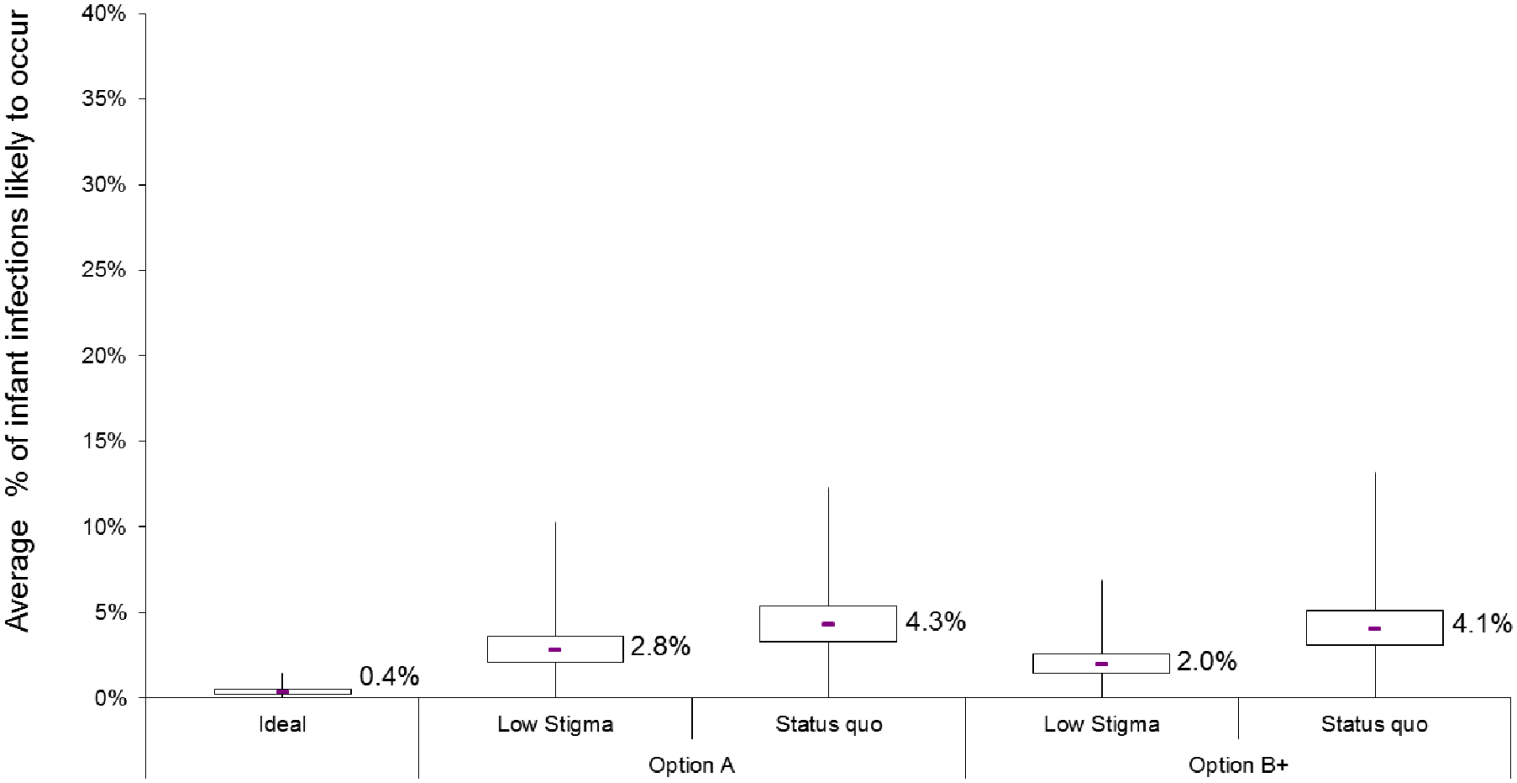
Results from the uncertainty analysis for Option A (red) and Option B+ (blue) scenarios and the idealised (green) scenario. The box plots show variation in the cohort-median mother-to-child transmission rates over 10,000 simulated cohorts. For each scenario, the box denotes the interquartile range (IQR: 25th to 75th percentiles) and the middle line denotes the median (50th percentile). **Whiskers capture values up to twice the width of the IQR, while those exceeding this** are shown as outliers (blue scattered tail).

The results of our uncertainty analysis, in which we varied both transmission rates and scenario probabilities, are presented in Fig 7. Each boxplot corresponds to a model scenario (for Option A or Option B+), and depicts variation in the average mother-to-child transmission rate likely to occur. As expected, there is considerable variation in the average percentage of infections estimated to occur in each of the scenarios. This reflects the compounding effect of uncertainty through the cascade.

For the ideal scenario, the interquartile range for the average mother-to-child transmission rate is 0.2% to 0.5%. For a low-stigma scenario, the model estimates a median of 2.0% (Interquartile range [IQR] 1.5–2.6%) of infants infected if the Option B+ programme had been in place in 2010, versus 2.8% (IQR 2.1–3.6%) of infections under the Option A programme. The median percentage of infants infected in the status quo scenario is 4.3% (IQR 3.3–5.4%) for Option A and 4.1% (IQR3.1–5.1%) for Option B+. +. This means that under the current B+ programme, assuming a similar cascade trajectory in 2017 as in 2010, eradicating stigma could reduce infant infections by approximately half (51%), according to our model estimates.

## Discussion

Stigma and discrimination are recognized barriers to all aspects of the HIV response[8–12], and while there is a growing body of evidence of the negative effects of stigma, there is still little routine or consistent data being collected on either the prevalence of stigma or their impact on specific HIV outcomes and the benefits that might derive from a more coordinated and scaled stigma-reduction response. This exploratory, proof of concept exercise, took one key HIV outcome—the average mother-to-child transmission rate—and demonstrated how modeling can provide important insights into the potential size of the impact of stigma.

The first insight provided by the model is that, due to the conditional probabilities and cumulative nature of the PMTCT cascade, and that stigma affects every stage of the cascade, reducing stigma can lead to large reductions in mother-to-child transmission rates. Examples of the potential impact of stigma reduction include increasing testing rates, improving drug initiation and adherence and/or improving all other risk-reduction activities. The effect of stigma is surprisingly large because stigma acts at every stage of the "cascade", and its effect is therefore cumulative through the cascade. Reducing stigma has a surprisingly large payoff, even comparable to biomedical interventions in which impact is related to an individual’s ability to adhere to treatment.

The model results indicate that, in a setting such as Johannesburg, under the previous Option A regime, about 35% of all infant infections could be averted, by successfully eradicating stigma, whilst this is higher at 51% of infections under Option B+. Given that data for parameterizing this model was extracted from the District Health Information System (DHIS) at the time that the 2010 guidelines were in place, the model follows these guidelines, and in addition we model the hypothetical case of assuming Option B+ steps had been in place. This highlights that as health-systems strengthen, delivery of care improves, and treatment is more effective—the relative impact of stigma becomes even greater, because reducing stigma matters the most where it causes women to “miss out” on the best treatment regimens. Indeed, our findings agree with that of Watts at al. (2010), who showed that in a high versus low functioning health setting, 53% versus 26% of infant infections could be due to stigma respectively[17].

### Strengths and limitations

The median transmission probabilities in each scenario for Option A are 4.3% (status quo), 2.8% (low stigma, and 0.4% (ideal), but variability in transmission within scenarios is larger than the differences in transmission between scenarios. Variation in the precision of the estimated values limits the precision of our estimates of the size of the effect of stigma on flow through the cascade. As with the cumulative effect of stigma on each step of the cascade, imprecision in estimates of parameters at each step of the cascade also multiplies through the cascade. Accurate estimation of the effect of stigma requires precise estimates of the model parameters (stigma, non-stigma and transmission through the cascade steps). Empirical data, particularly on stigma, is still scarce, posing an important limitation on the model. However, the results presented here are meaningful to the extent that these estimates are plausible, since the data were acquired from a viable source. In addition, we used the same transmission probabilities across each scenario, and so the relative effect of stigma and impact of eliminating stigma should not be greatly affected by this limitation.

Data were extracted from a study carried out in 2010, which may represent lower retention levels within the PMTCT cascade as compared to 2017, with additional investment and political will strengthening both health systems and the drive to achieve elimination of mother-to-child transmission[15]. In this respect, the current model projections may underestimate the impact of stigma, since the strengthening of health-systems would reduce supply-side barriers, and increase the proportion of infections attributed to stigma (although we would expect an over-all decline in the total number of infections).

Despite the uncertainty of the model estimates, the result holds firm that the effect of reducing stigma could be very effective at reducing vertical transmission. Other modeling studies have suggested that the elimination target of less than 2% HIV transmission is achievable, however estimates are based on the uptake of PMTCT and biological effects, without modelling the potential impact of stigma on achieving this goal [40]. Our model suggests that 4% transmission occurs even with Option B+ in the status quo scenario, but this reduces to the 2% transmission target if stigma is eradicated. These examples highlights how the exclusion of structural factors in models, such as stigma may lead to over-optimistic model estimates. Cascade models, offer the opportunity to develop an integrated framework within transmission models which address both the limitations of supply and demand-side factors as well as adherence, to make estimates closer to what is plausibly achievable.

### Implications for policy and practice

Similar to Watts et al. (2010), our model projections demonstrate how the negative effect of stigma on retention of women across the PMTCT cascade can be relatively small at any given stage of the cascade but combines throughout the stages overall [31]. Cumulatively, the effect of stigma increasing the number of new infant HIV infections can be substantial. The cumulative nature of the PMTCT cascade can be harnessed for efficiency in investment by prioritizing interventions that can affect multiple stages of the cascade simultaneously, the most obvious of which are interventions to reduce stigma directly[74]. Yet, there is a paucity of stigma-reduction specific intervention programmes that focus primarily on PMTCT, despite multiple studies that highlight stigma as a key barrier to care[18]. However, a recent systematic review assessing the impact more generally of stigma interventions, shows the breath of such interventions in targeting community, individual, organizational, interpersonal and public policy levels, highlighting the importance of targeting both the drivers and manifestations of stigma concurrently[13]. There is also a growing number of HIV stigma-reduction interventions targeting health facilities more broadly, with a focus on all health facility staff and institutional structures, as opposed to just those services providing care to pregnant women.

While this work cannot replace empirical measurement of causal effect of stigma; given the paucity of such measurements, it allows us to at least demonstrate the potential magnitude of the effect. The exact numbers estimated by our model should be interpreted cautiously and from a qualitative rather than a quantitative perspective. Our findings indicate that reducing stigma may be critical in reaching virtual MTCT elimination targets. This will be particularly relevant as countries improve supply-side factors, which will make the relative impact of stigma even greater.

Stigma continues to feature prominently in global, country and sub-national discourse as an enduring barrier to attaining epidemic control, yet few resources are devoted to measuring and addressing them. In the absence of empirical data on stigma, modeling offers an important, if imprecise, starting point to begin grappling more directly with this continuing and potentially large gap in the HIV response. In not addressing stigma more aggressively because of lack of empirical data or the inherent challenge in measuring effect sizes of stigma-reduction—when the effect may nevertheless be both large and pervasive—we may be missing a fundamental piece of the HIV response.

## Supporting information

S1 File Details on literature search and Johannesburg DHIS. (DOCX)

S1 Table Summary of stigma-related barriers. (DOCX)

S2 Table Summary of non-stigma-related barriers. (DOCX)
