# Supplementary material for: Can mother-to-child transmission of HIV be eliminated without addressing the issue of stigma? Modeling the case for a setting in South Africa
Source: PLoS One. 2017 Dec 8;12(12):e0189079. doi: 10.1371/journal.pone.0189079 (PMC5722282; doi:10.1371/journal.pone.0189079)
Supplement: S1 File — (DOCX) [file pone.0189079.s001.docx]

**S1 File: Details on literature search and Johannesburg DHIS**

Searches for the literature review were conducted with PubMed and Google Scholar using these combined search terms: PMTCT or mother-to-child transmission with stigma, discrimination, retention, adherence, drop out, and participation. A search for grey literature was conducted using similar terms on the Web sites of such multilateral organizations as UNAIDS and WHO; such bilateral organizations as DFID and the United States *President's Emergency Plan for AIDS Relief* (PEPFAR); and such international non-profit organizations as FHI360 and Elizabeth Glaser Paediatric AIDS Foundation. The references sections of all articles found were consulted for additional relevant literature. A total of 111 articles were reviewed.

Data from the DHIS database were accessed by the monitoring and evaluation team at the Wits Reproductive Health and HIV Institute (WRHI), in Johannesburg, South Africa, from July 5–16, 2011. To help us understand the database, key informants at the WRHI provided background information before data were accessed. Data from three provincial hospitals and 17 ANC clinics, primary healthcare centers, postnatal clinics, and immunization clinics located in the city of Johannesburg were accessed. Input parameters were collected from April 2010, when the new PMTCT guidelines were implemented, until May 2011.
