# Supplementary material for: Can mother-to-child transmission of HIV be eliminated without addressing the issue of stigma? Modeling the case for a setting in South Africa
Source: PLoS One. 2017 Dec 8;12(12):e0189079. doi: 10.1371/journal.pone.0189079 (PMC5722282; doi:10.1371/journal.pone.0189079)
Supplement: S1 Table — (DOCX) [file pone.0189079.s002.docx]

**S1 Table. Summary of stigma-related barriers**

| Cascade Stage | Description | Potential Reasons for Stigma Acting as a Barrier | Sources |
| --- | --- | --- | --- |
| 1 | % of pregnant women accessing ANC services | Fear of being offered an HIV test–despite opt-out testing  Anticipated stigma from refusing a test or from testing positive | (1-4) |
| 2 | % of pregnant women who are offered and accept HIV test and receive results | Fear of being found positive  Fear of disclosure of test results  Lack of partner involvement or permission to test  Feeling that one could not deal with a positive result | (5-10) |
| 3  AZT and HAART | % of pregnant women who know HIV status initiated on AZT and HAART | Fear of unwanted disclosure of positive status  Fear of negative repercussions from family and community if status is discovered  Fear of domestic violence  Community-driven mistrust of ART | (11-13) (14) |
| 3  AZT and HAART | % of women who adhere to treatment through pregnancy, labor, and delivery | Fear of unwanted disclosure of positive status  Fear of negative repercussions from family and community if status is discovered  Fear of domestic violence  Nonadherence when friends and family are present | (15) |
| 4  AZT | % of women who give birth in a hospital or birthing center with trained healthcare assistants | Fear of stigmatizing attitudes from staff at the maternity ward  Nondisclosure of status may prevent necessary support from family/spouse to deliver at a healthcare facility | (4, 9) |
| 4  AZT | % of women on AZT who receive correct medication at labor ward and,  % of women of unknown HIV status or who are HIV positive, who have not received any treatment prior to admission | Fearing stigma, a woman may not disclose HIV status at delivery, and a test may not be performed  Nondisclosure out of shame for not having received PMTCT treatment prior to labor and delivery | (16) |
| 5  AZT and HAART | % of infants whose mothers receive 6 weeks of NVP for their infants | As packaging is identifiable, mothers may refuse to receive or leave behind infant NVP syrup, fearing it will disclose her status. | (1) |
| 5  AZT and HAART | % of infants who adhere to the NVP treatment for first 6 weeks post-delivery | Fearing family or community will see her delivering the NVP syrup, a mother may not adhere to avoid disclosure | (1) |
| 6  AZT and HAART | % of women who exclusively breastfeed for 6 months | Fear that not following cultural infant feeding norms will disclose HIV status  Shame from possible HIV transmission via breastfeeding | (17-20) |

**Reference**

1. Laher F, Cescon A, Lazarus E, Kaida A, Makongoza M, Hogg R, et al. Conversations with mothers: Exploring reasons for prevention of mother-to-child transmission (PMTCT) failures in the era of programmatic scale-up in Soweto, South Africa. AIDS & Behavior. 2011.

2. Rahangdale L, Banandur P, Sreenivas A, Turan JM, Washington R, Cohen CR. Stigma as experienced by women accessing prevention of parent-to-child transmission of HIV services in Karnataka, India. AIDS Care. 2010;22(7):836 — 42.

3. Nguyen TA, Oosterhoff P, Pham YN, Hardon A, Wright P. Health workers' views on quality of prevention of mother-to-child transmission and postnatal care for HIV-infected women and their children. Human Resources for Health. 2009;7(39).

4. Ujiji OA, Ekström AM, Ilako F, Indalo D, Wamalwa D, B. R. Reasoning and deciding PMTCT-adherence during pregnancy among women living with HIV in Kenya. Culture, Health & Sexuality. 2011;13(7):829-40.

5. Rakoasi S. HIV counselling and testing of pregnant women attending antenatal clinics in Botswana. Journal of Health Population and Nutrition. 2005(1):58-65.

6. Turan JM, Miller S, Bukusi EA, Sande J, Cohen CR. HIV/AIDS and maternity care in Kenya: How fears of stigma and discrimination affect uptake and provision of labor and delivery services. AIDS Care. 2008;20(8):938 - 45.

7. Creek T, Ntumy R, Mazhani L, Moore J, Smith M, Han G, et al. Factors associated with low early uptake of a national program to prevent mother to child transmission of HIV (PMTCT): Results of a survey of mothers and providers, Botswana, 2003. AIDS & Behavior. 2009(13):356-64.

8. Sprague C, Chersich MF, Black V. Health system weaknesses constrain access to PMTCT and maternal HIV services in South Africa: A qualitative enquiry. AIDS Research & Therapy. 2011;8(10):1-9.

9. Turan JM, Bukusi EA, Onono M, Holzemer WL, Miller S, Cohen CR. HIV/AIDS stigma and refusal of HIV testing among pregnant women in rural Kenya: Results from the MAMAS study. AIDS & Behavior. 2011;15(6):1111-20.

10. Oosterhoff P, Hardon AP, Nguyen TA, Pham NY, Wright P. Dealing with a positive result: Routine HIV testing of pregnant women in Vietnam. . AIDS Care. 2008;20(6):654 - 9.

11. Duff P, Kipp W, Wild TC, Rubaale T, Okech-Ojony J. Barriers to accessing highly active antiretroviral therapy by HIV-positive women attending an antenatal clinic in a regional hospital in western Uganda. Journal of the International AIDS Society. 2010;13(37):1-9.

12. Kunihira NR, Nuwaha F, Mayanja R, Peterson S. Barriers to use of antiretroviral drugs in Rakai District of Uganda. African Health Sciences. 2010;10(2):120 - 9.

13. Mepham S, Zondi Z, Mbuyazi A, Mkhwanazi N, Newell ML. Challenges in PMTCT antiretroviral adherence in northern KwaZulu-Natal, South Africa. AIDS Care. 2011;23 (6):741-7.

14. Nzaumvila DK, Mabuza LH. Why do women not return for CD4 count results at Embhuleni Hospital, Mpumalanga, South Africa? curationis. 2015;38(1):1-8.

15. Duff P, Walter K, Wild T, Rubaale T, Okech-Ojony J. Barriers to accessing highly active antiretroviral therapy by HIV-positive women attending an antenatal clinic in a regional hospital in western Uganda. Journal of the International AIDS Society. 2010;13(37).

16. Otieno PA, Kohler PK, Bosire RK, Brown ER, Macharia SW, John-Stewart GC. Determinants of failure to access care in mothers referred to HIV treatment programs in Nairobi, Kenya. AIDS Care 2010 22(6):729 - 36.

17. Sibeko L, Coutsoudis A, Nzuza S, Gray-Donald K. Mothers’ infant feeding experiences: Constraints and supports for optimal feeding in an HIV-impacted urban community in South Africa. Public Health Nutrition. 2007;12(11):1983-90.

18. Desclaux A, Alfieri C. Counseling and choosing between infant-feeding options: Overall limits and local interpretations by health care providers and women living with HIV in resource-poor countries (Burkina Faso, Cambodia, Cameroon). Social Science & Medicine. 2009;69:821-9.

19. Morgan MC, Masaba RO, Nyikuri M, Thomas TK. Factors affecting breastfeeding cessation after discontinuation of antiretroviral therapy to prevent mother-to-child transmission of H.I.V. AIDS Care. 2010;22:866-73.

20. Onono MA, Carraher N, Cohen RC, Bukusi EA, Turan J. Use of personal digital assistants for data collection in a multi-site AIDS stigma study in rural South Nyanza, Kenya. African Health Sciences. 2011;11(3):464-73.
