# Supplementary material for: Can mother-to-child transmission of HIV be eliminated without addressing the issue of stigma? Modeling the case for a setting in South Africa
Source: PLoS One. 2017 Dec 8;12(12):e0189079. doi: 10.1371/journal.pone.0189079 (PMC5722282; doi:10.1371/journal.pone.0189079)
Supplement: S2 Table — (DOCX) [file pone.0189079.s003.docx]

**S2 Table. Summary of non-stigma-related barriers.**

| Cascade Stage | Description | Potential Health System Delivery Barriers and Non-stigma-related Access Barriers | Sources |
| --- | --- | --- | --- |
| 1 | % of pregnant women accessing ANC services | Transportation and time costs associated with travel  Distance to the ANC facility  Waiting times and a shortage of staff at the ANC clinic | (1, 2) |
| 2 | % of pregnant women who are offered and accept HIV test and receive results | Availability of testing supplies  Overworked staff may not have time to provide thorough counseling  Lack of mother’s understanding of PMTCT and its importance  Women tested but fail to receive test results due to confusion or poor follow-up | (1, 3-6) |
| 3  AZT and HAART | % of pregnant women who know HIV status initiated on AZT and HAART | Transportation and time costs associated with receiving medication  Fear of consequences from imperfect adherence  Fear of side effects  Interrupted supplies and stockout  Poor or non-existent CD4 testing capabilities | (1, 7-12) |
| 3  AZT and HAART | % of women who adhere to treatment through pregnancy, labor, and delivery | Theft of ART drugs by friends and family  Transportation and time costs associated with getting medicine once initial doses are exhausted  Fear of or experienced side effects  Interrupted supplies and stockout | (10, 12-15) |
| 4  AZT | % of women who give birth in a hospital or birthing center with trained healthcare assistants | Transportation costs  Community, family, personal desire to have a home birth  Lack of understanding of the importance of delivery in a health facility for PMTCT | (2, 12, 16) |
| 4  AZT | % of women on AZT who receive correct medication at labor ward, and  % of women of unknown HIV status or who are HIV positive, who have not received any treatment prior to admission | Supply interruption and stockout  Overworked delivery wards  Complications and emergency conditions will trump PMTCT precautions  Too little time for full PMTCT treatment before delivery  Too little time for a rapid test for women whose status is unknown | (17) |
| 5  AZT and HAART | % of infants whose mothers receive 6 weeks of NVP for their infants | Supply interruptions and stockout  Difficulty in returning to a supply center if NVP syrup is spilled or lost, or if an inadequate supply was given initially | (12) |
| 5  AZT and HAART | % of infants who adhere to the NVP treatment for 6 weeks post-delivery | Spillage of NVP syrup  Infant spits up syrup and mother chooses to stop administering treatment  Side-effects or adverse reactions | (12) |
| 6  AZT and HAART | % of women who exclusively breastfeed for 6 months | Work or travel away from the infant may prevent exclusive breastfeeding  Changes to feeding guidelines in the past may confuse a mother about duration and importance of exclusive breastfeeding  Healthcare workers may steer a woman toward their own feeding preferences  A caregiver apart from the mother may introduce the infant to other foods or liquids without the mother’s consent or knowledge | (1, 12, 18, 19) |

**References**

1. Sprague C, Chersich MF, Black V. Health system weaknesses constrain access to PMTCT and maternal HIV services in South Africa: A qualitative enquiry. AIDS Research & Therapy. 2011;8(10):1-9.

2. Kasenga F, Byass P, Emmelin M, Hurtig AK. The implications of policy changes on the uptake of a PMTCT programme in rural Malawi: First three years of experience. Global Health Action. 2009;2:1-7.

3. Creek T, Ntumy R, Mazhani L, Moore J, Smith M, Han G, et al. Factors associated with low early uptake of a national program to prevent mother to child transmission of HIV (PMTCT): Results of a survey of mothers and providers, Botswana, 2003. AIDS & Behavior. 2009(13):356-64.

4. Orie EF, Songca PP, Moodley J. An audit of PMTCT services at a regional hospital in South Africa. South African Family Practice. 2009;51(6):492-5.

5. Balcha TT, Lecerof SS, Jeppson AR. Strategic challenges of PMTCT program implementation in Ethiopia. Journal of the International Association of Physicians in AIDS Care. 2011;10(3):187-92.

6. Moses AE, Chama C, Udo SM, Omotora BA. Knowledge, attitude and practice of ante-natal attendees toward prevention of mother to child transmission (PMTCT) of HIV infection in a tertiary health facility, northeast Nigeria. Internet Journal of Third World Medicine. 2009;8(1):9-.

7. Mepham S, Zondi Z, Mbuyazi A, Mkhwanazi N, Newell ML. Challenges in PMTCT antiretroviral adherence in northern KwaZulu-Natal, South Africa. AIDS Care. 2011;23 (6):741-7.

8. Mekonnen G. Factors influencing utilization of PMTCT services in Addis Ababa, Ethiopia. Amsterdam: KIT (Royal Tropical Institute)/Vrije Universiteit Amsterdam; 2009.

9. Duff P, Kipp W, Wild TC, Rubaale T, Okech-Ojony J. Barriers to accessing highly active antiretroviral therapy by HIV-positive women attending an antenatal clinic in a regional hospital in western Uganda. Journal of the International AIDS Society. 2010;13(37):1-9.

10. Horwood C, Haskins L, Vermaak K, Phakathi S, Subbaye R, Doherty T. Prevention of mother to child transmission of HIV (PMTCT) programme in KwaZulu-Natal, South Africa: An evaluation of PMTCT implementation and integration into routine maternal, child and women’s health services. Tropical Medicine and International Health,. 2010;15(9):992-9.

11. Kunihira NR, Nuwaha F, Mayanja R, Peterson S. Barriers to use of antiretroviral drugs in Rakai District of Uganda. African Health Sciences. 2010;10(2):120 - 9.

12. Laher F, Cescon A, Lazarus E, Kaida A, Makongoza M, Hogg R, et al. Conversations with mothers: Exploring reasons for prevention of mother-to-child transmission (PMTCT) failures in the era of programmatic scale-up in Soweto, South Africa. AIDS & Behavior. 2011.

13. Duff P, Walter K, Wild T, Rubaale T, Okech-Ojony J. Barriers to accessing highly active antiretroviral therapy by HIV-positive women attending an antenatal clinic in a regional hospital in western Uganda. Journal of the International AIDS Society. 2010;13(37).

14. Igwegbe AO, Ugboaja JO, Nwajiaku LA. Prevelance and determinants of non-adherence to antiretroviral therapy among HIV-positive pregnant women in Nnewi, Nigeria. International Journal of Medicine and Medical Sciences. 2010;2(8):238-45.

15. El-Khatib Z, Ekstrom AM, Coovadia A, Abrams EJ, Petzold M, Katzenstein D, et al. Adherence and virologic suppression during the first 24 weeks on antiretroviral therapy among women in Johannesburg, South Africa - A prospective cohort study. BMC Public Health. 2011;11(88):1-13.

16. Youngleson M, Nkurunziza P, Jennings K, Arendse J, Mate K, Barker P. Improving a mother to child HIV transmission programme through health system redesign: Quality improvements, protocol adjustments, and resource addition. PLoS One. 2010;5(11):1-8.

17. Pai NP, Klein MB. Improving a mother to child HIV transmission programme through health system redesign: Quality improvements, protocol adjustments, and resource addition. Women's Health. 2009;5(1):55-62.

18. Sibeko L, Coutsoudis A, Nzuza S, Gray-Donald K. Mothers’ infant feeding experiences: Constraints and supports for optimal feeding in an HIV-impacted urban community in South Africa. Public Health Nutrition. 2007;12(11):1983-90.

19. van Lettow M, Bedell R, Landes M, Gawa L, Gatto S, Mayuni I, et al. Uptake and outcomes of a prevention-of mother-to-child transmission (PMTCT) program in Zomba District, Malawi. BMC Public Health. 2011;11(426).
